# Diversity and functional prediction of fungal communities in different segments of mongolian horse gastrointestinal tracts

**DOI:** 10.1186/s12866-023-03001-w

**Published:** 2023-09-09

**Authors:** Yiping Zhao, Xiujuan Ren, Haiqing Wu, He Hu, Chao Cheng, Ming Du, Yao Huang, Xiaoqing Zhao, Liwei Wang, Liuxi Yi, Jinshan Tao, Yajing Li, Yanan Lin, Shaofeng Su, Manglai Dugarjaviin

**Affiliations:** 1 College of Animal Science, Inner Mongolia Key Laboratory of Equine Genetics, Breeding and Reproduction, Scientific Observing and Experimental Station of Equine Genetics, Breeding and Reproduction, Ministry of Agriculture and Rural Affairs, Equine Research Centre, Inner Mongolia Agricultural University, Hohhot, 010018 China; 2 Inner Mongolia Academy of Agricultural and Animal Husbandry Sciences, Biotechnology Research Centre, Hohhot, 010031 China; 3 College of Life Science and Technology, Jining Normal University, Ulanqab, 012000 China; 4 Education Department, Baotou Light Industry Vocational Technical College, Baotou, China

**Keywords:** Fungi, ITS1, High-throughput sequencing, Intestinal microflora, Mongolian horses

## Abstract

**Background:**

Anaerobic fungi are effective fibre-degrading microorganisms in the digestive tract of horses. However, our understanding of their diversity and community structure is limited, especially in different parts of the gastrointestinal tract.

**Results:**

For the first time, high-throughput sequencing technology was used to analyse and predict fungal microbial diversity in different parts of the gastrointestinal tract of Mongolian horses. The results revealed that the richness and diversity of fungi in the hindgut of Mongolian horses were much higher than those in the foregut. The foregut was dominated by Basidiomycota and Ascomycota, whereas the hindgut was dominated by Neocallimastigomycota and Basidiomycota. At the genus level, the relative abundance of many pathogenic fungi (*Cryptococcus, Cladosporium, Alternaria*, and *Sarocladium*) in the foregut was significantly higher than that in the posterior gut, indicating that Mongolian horses have strong disease resistance. The prediction of fungal function also showed significant differences in the fungal flora between the foregut and the hindgut. The fungi in Mongolian horses’ foreguts were mainly pathologically nutritive and contained many animal and plant pathogens, particularly in the small intestine (jejunum and ileum). This indicates that the foregut may be the most important immune site in the digestive system of Mongolian horses, which explains the high disease resistance of Mongolian horses. The number of unassigned functional groups in the posterior gut was significantly higher than that in the anterior gut, indicating that the functions of fungal groups in the posterior gut have not been fully explored, and further studies are required in the future.

**Conclusions:**

Analysis of high-throughput sequencing results revealed that the fungal composition varied greatly among different gastrointestinal tract segments in Mongolian horses, whose hindgut contains many anaerobic fungi involved in plant cellulose degradation. This provides important basic data for studying fungal diversity in the digestive system of healthy horses, which can be used for the health assessment of horses and provides clues for further research on the disease resistance and digestive capacity of horses, as well as a reference for the early diagnosis of intestinal diseases and innovative treatment methods.

**Supplementary Information:**

The online version contains supplementary material available at 10.1186/s12866-023-03001-w.

## Background

The gastrointestinal tract (GIT) microbiota not only provides the host with nutrients such as amino acids, vitamins, and short-chain fatty acids that are necessary for growth but also plays important roles in digestion, absorption, metabolism, and the immune system [1, 2]. However, the stability of the GIT microbiota is easily influenced by intrinsic (host) and extrinsic (environmental) factors [3], such as species [4], age [5], geographic location [6], transportation [7, 8], exercise stress [8], diet [9], and season [10]. Impairment or dysregulation of the equine GIT and its microbiome may lead to pathological responses [10–12]. Studies have revealed that diarrhoea in horses is closely related to changes in the composition and structure of intestinal microbiota [13, 14]. In addition, alterations in the gut microbiome may lead to various diseases, such as obesity [15], laminitis [16], equine glandular gastric disease (EGGD) [17], colic [18], and colitis [19]. Consequently, it is essential to determine the gut microbiota composition in healthy horses to determine the effects of metabolic disorders and diseases.

Horses are classified as monogastric herbivores, and they have a specific GIT tissue, and a strong posterior intestinal system. Studies have demonstrated that the equine GIT can be divided into two main regions: the upper GIT (UG) and lower GIT (LG), and each segment of the GIT has a different ecosystem. The bacterial microbiota composition of adjacent compartments is more similar than that of far-apart compartments [20]. The microbiota structure of equine UG (stomach, jejunum, and ileum) is more complex and diverse because of the influence of many environmental bacteria in forage. In contrast, the microbiota composition of the lower equine gut (caecum and colon) appears to be relatively stable, despite variables such as individual history, breed, or age [20–22]. The equine GIT contains various microbial communities, including fungi, parasites, protozoa, archaea, viruses, and bacteria [23]. However, knowledge about the role of fungi in the GIT and their contribution to the microbiome of healthy horses is limited, as reflected in only a few studies on hindgut anaerobic fungi [24]. Anaerobic fungi (Neocallimastigomycota) are widely found in the hindgut of horses and play an important role in degrading plant cellulose, given that they possess a complete and highly effective group of plant cell wall-degrading enzymes [25]. To date, anaerobic fungi isolation and morphological identification mainly rely on purification culture and microscopy. Nine genera have been identified, and more than 20 species have been described [26]. Studies have indicated that the digestive tract of horses is dominated by novel, as-yet-uncultured anaerobic fungi that are different from previously described foregut herbivores [24].

In horses, the gut microbiota has been used as a molecular marker for bacteria and archaea, mainly by DNA sequencing, targeting variable regions of the 16 S rRNA gene. In contrast, eukaryotic communities (e.g., anaerobic fungi) have been described in the equine hindgut and have been identified to play an important role in digestion [11, 13]. Next-generation sequencing (NGS) technology has been applied to analyse fungal diversity copies in equine gut contents and faecal samples, but relevant research remains far from adequate [14, 24]. Indeed, stool reflects microbial changes only in the distal portion of the hindgut and not the entire GI microbiota. Therefore, evaluating the composition of anaerobic fungal communities in different parts of the gastrointestinal tract is important. This study aimed to characterize and compare the fungal composition in different parts of the gastrointestinal tract of Mongolian horses using NGS technology.

## Materials and methods

### Horse and sample collection

GIT content samples were obtained from five healthy Mongolian horses (three males and two females, mean age 4.4 years, 3–6 years, weight 292.8 ± 11.9 kg) that were euthanized by overdose of pentobarbital sodium **(**Table 1**)**. As described in a previous study [20], these horses were fed on the same pasture in the grasslands of the Xilin Gol League, Inner Mongolia, China, maintaining the same grazing conditions without any history of intestinal diseases. The gastrointestinal sample collection sites included the stomach (S), jejunum (J), and ileum (I) for the upper GIT and the caecum (C), ventral colon (VC), and dorsal colon (DC) for the lower GIT. The intestinal segments were tied to prevent mixing between adjacent segments, and the contents were collected in the middle part of each segment after horizontal placement. Samples were collected in 50 mL sterile enzyme-free centrifuge tubes, snap-frozen in liquid nitrogen, and stored at − 80 °C for further study.

**Table 1:** Specific details of the experimental Mongolian horses

Horse sample	Age	Sex	Weight (kg)	Reason for euthanasia	Feeding
H1	3	F	275	Neurological	Grass
H2	3	M	296	Old wound	Grass
H3	5	M	298	Navicular disease	Grass
H4	5	F	285	Osteoarthritis	Grass
H5	6	M	310	Old wound	Grass

### DNA extraction, PCR amplification, and library construction

Genomic DNA was extracted from the GIT samples by the cetyltrimethylammonium bromide (CTAB) method [27], and the concentration and purity of DNA were detected by 1% agarose gel electrophoresis. DNA diluted at 1 ng/µL was used as a template for PCR amplification using specific ITS1 region primers: 1737 F (5’-GGAAGTAAAAGTCGTAACAAGG-3’) and 2043 R (5’-GCTGCGTTCTTCATCGATGC-3’) [28, 29]. Fungal genomic library construction and on-machine sequencing were completed by Beijing Nuohe Zhiyuan Technology Co., Ltd. An Ion Plus Fragment Library Kit (No: 4471252, Thermo, USA) was used to construct the library. After Qubit quantification and library qualification, the constructed library was sequenced by the Ion S5^TM^XL platform.

### Bioinformatics analysis

#### Sequencing data processing

Cutadapt (V1.9.1, http://cutadapt.readthedocs.io/en/stable/) was used for preliminary quality control to obtain raw reads, which were compared with the Unite database (https://unite.ut.ee/) to remove chimeric sequences and obtain clean reads [30–32].

#### OTU clustering and species annotation

Clean reads were clustered using UPARSE software (v7.0.1001, http://drive5.com/uparse/), and sequences were clustered into operational taxonomic units (OTUs) based on a 97% identity threshold to obtain the number of OTUs [33]. The OTU representative sequences were analysed by species annotation using the BLAST method in QIIME software (Version 1.9.1, http://qiime.org/scripts/assign_taxonomy.html) and the UNITE database [34, 35]. Finally, the data of each sample were homogenized for subsequent alpha and beta diversity analysis.

#### Alpha diversity analysis

QIIME software (Version 1.9.1) was used to calculate the ACE, Chao1, Shannon, observed-species, and Goods-coverage indices. R software (Version 2.15.3) was used to draw the dilution curve, rank abundance curve, and species accumulation curve and to analyse the differences in the alpha diversity index between groups. Nonmetric multidimensional scaling (NMDS) analysis was performed with the vegan package using R software (Version 2.15.3).

#### Beta diversity analysis

QIIME software (Version 1.9.1) was used to calculate the UniFrac distance and construct the UPGMA clustering tree. Linear discriminant analysis effect size (LEfSe) analysis was conducted using LEfSe software, and the default screening value of the LDA score was set to 4. LEfSe analysis was used to analyse different species between groups. FunGuild software was used to predict the functional composition of the fungal communities [36].

### Data analysis

All parameters of different segments of the equine GIT are expressed as the mean ± standard deviation. Statistical significance was analysed using ANOVA in SPSS 22.0 software, and multiple comparisons were performed by the LSD test. A P value < 0.05 indicated a significant difference, and a P value < 0.01 indicated a highly significant difference.

## Results

### Sequencing results were clustered with OTUs

**Fig. 1:**
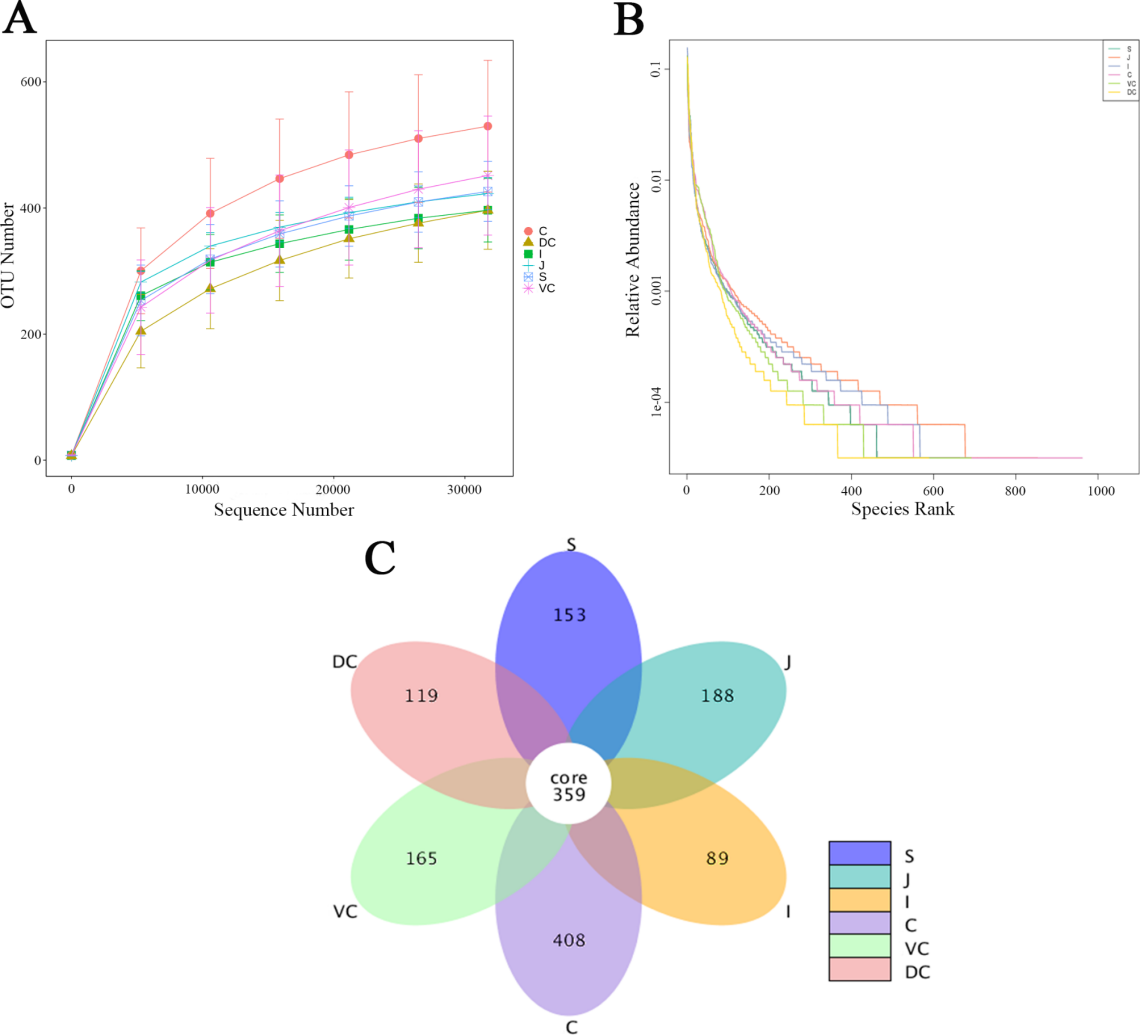
Microbial community diversity in the gastrointestinal tract of Mongolian horses. **A**: Dilution curve; **B**: Rank abundance curve; **C**: OTU Venn diagram of gastrointestinal flora. In each figure, S represents the stomach segment, J represents the jejunum segment, I represents the ileum segment, C represents the caecum segment, VC represents the ventral colon segment, and DC represents the dorsal colon segment

In this study, the fungal diversity in the GIT of Mongolian horses was completely consistent with that previously reported, which provided a reference for further analysis [20]. After the sequencing data of 30 content samples from different compartments of equine GIT were spliced, filtered, and screened, the average number of valid sequences was 74,477, and the average number of reads of constructed OTUs with annotation information was 58,739. The average number of OTUs was 525 (Additional File 1). Dilution curve analysis showed that the sequencing depth of all samples in different sections almost reached a plateau at the OTU level, indicating that the current sequencing depth covered most microorganisms, and the amount of undetected data had little impact on subsequent analysis. This curve also indirectly reflected the species richness in different segments of the GI tract; the cecum (C) exhibited greater species richness than other parts **(**Fig. 1A**)**. The rank abundance curve can objectively reflect the richness and evenness of species in the sample [37], and the results showed that the species richness and evenness were similar in each segment of the GIT at the OTU level **(**Fig. 1B**)**.

The common and unique OTUs among different GIT segments were analysed based on the results of OTU cluster analysis. According to the petal diagram between different gastrointestinal segments, 359 OTUs were shared by the six groups of samples, which could be divided into six phyla **(**Fig. 1C, Additional File 2 and 3). A Venn plot of the lower GIT showed that the proportion of unique OTUs in the caecum, ventral colon, and dorsal colon in the total OTUs was 30.11%, 14.06%, and 10.20%, respectively, and the number of OTUs accounted for 27.11% of the total number of OTUs (Additional File 4).

### Alpha diversity analysis

At a sequencing depth of 31,744 reads per sample, the alpha diversity indices (ACE, Chao1, Shannon, observed-species, Good’s coverage) were calculated to analyse the diversity and richness of microbial communities within the samples **(**Table 2**)**. The richness indices were significantly higher (*P* < 0.05) in the caecum and ventral colon groups than in the small intestine group (jejunum and ileum) (observed species: 396.60 ± 56.35 (I) ~ 530.00 ± 116.83 (C), Chao1: 456.06 ± 77.84 (I) ~ 592.96 ± 130.23 (C), ACE: 458.39 ± 68.45 (I) ~ 600.33 ± 122.78 (C)). There was no significant difference in the species diversity index (Shannon: 5.17 ± 0.50 (I) ~ 5.28 ± 0.47 (C)). It is widely acknowledged that the Good’s coverage index can represent the coverage of the test results. The larger the index, the higher the coverage, and the more it represents the actual situation in the sample. The coverage index of fungal flora in all samples was above 99.6%, and the dilution curve tended to be flat, indicating that the amount of data was reasonable and could cover most species in the samples.

**Table 2:** Comparison of microbial diversity in different digestive parts of Mongolian horses

Sample	ACE	Chao1	Shannon	Observed species	Goods coverage
S	503.60 ± 58.14^Aa^	491.80 ± 51.70^ab^	5.09 ± 0.61	426.40 ± 53.14^b^	0.997 ± 0.001
J	477.84 ± 30.26^Ab^	475.04 ± 31.81^b^	5.41 ± 0.51	423.00 ± 28.39^b^	0.998 ± 0.000
I	458.39 ± 68.45^Bb^	456.06 ± 77.84^b^	5.17 ± 0.50	396.60 ± 56.35^b^	0.998 ± 0.000
C	600.33 ± 122.78^Aa^	592.96 ± 130.23^a^	5.28 ± 0.47	530.00 ± 116.83^a^	0.997 ± 0.001
VC	579.45 ± 105.23^Aa^	582.49 ± 126.52^ab^	5.17 ± 0.93	451.60 ± 105.67^ab^	0.996 ± 0.001
DC	501.68 ± 59.62^Aa^	490.72 ± 62.38^ab^	4.59 ± 1.05	396.60 ± 69.17^b^	0.997 ± 0.001

Notes: (1) Analysis of different samples under the threshold of 97% identity. (2) Different uppercase letters indicate significance at *P* < 0.01, different lowercase letters indicate significance at *P* < 0.05, and the same letters in the superscripts represent *P* > 0.05. S represents the stomach, J represents the jejunum, I represents the ileum, C represents the caecum, VC represents the ventral colon, and DC represents the dorsal colon

### Analysis of fungal composition structure based on OTUs

**Fig. 2:**
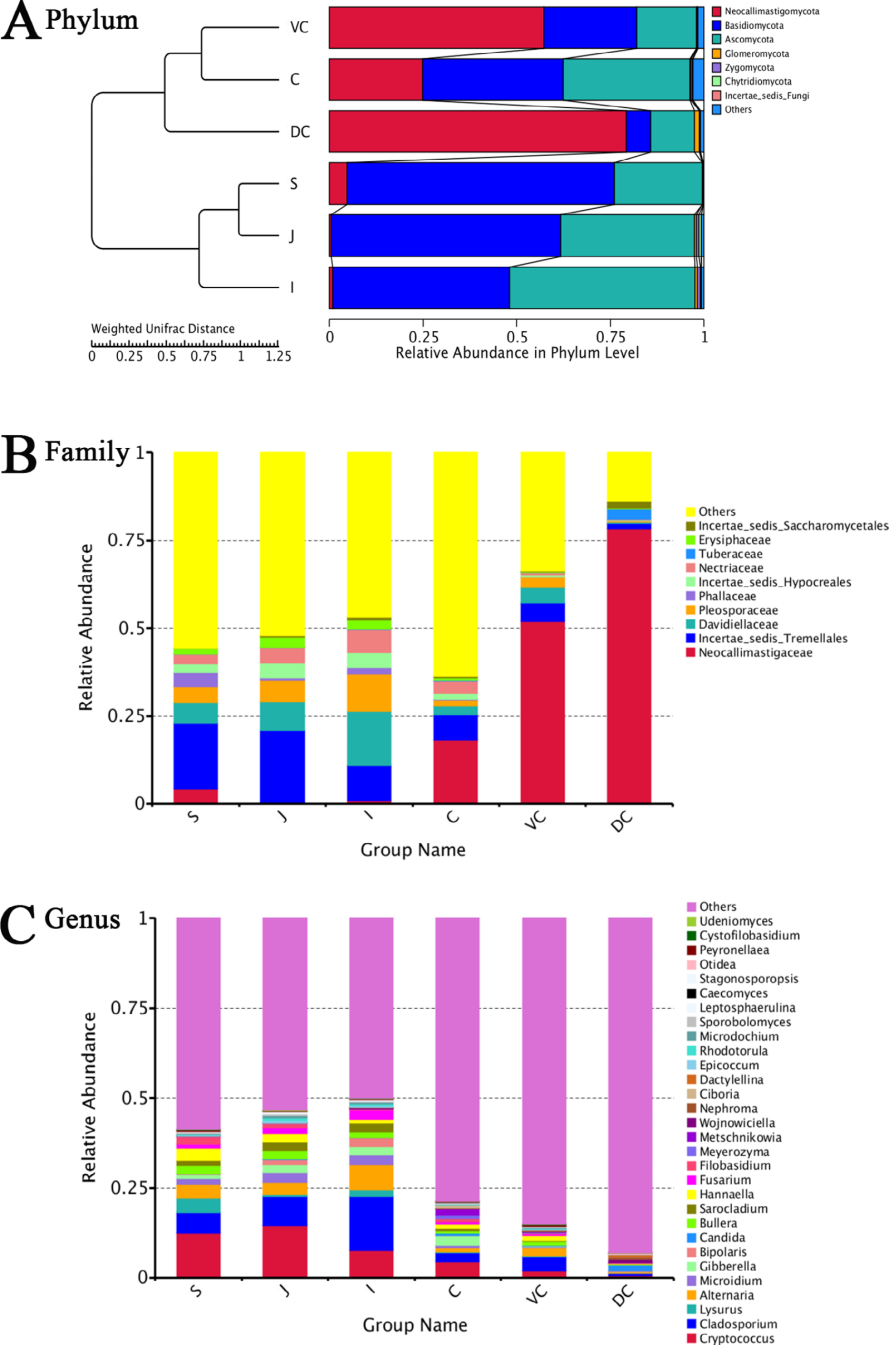
Relative abundance in luminal Mongolian horses GIT fungal composition. **A**: On the left is the UPGMA clustering tree structure, and on the right is the relative species abundance distribution of each sample at the phylum level; **B**: Subject level; **C**: Genus level. In each figure, S represents the stomach segment, J represents the jejunum segment, I represents the ileum segment, C represents the caecum segment, VC represents the ventral colon segment, and DC represents the dorsal colon segment

According to the OTU species annotation results, Basidiomycota and Ascomycota had the highest proportion in the foregut. The fungal composition of the hindgut contents changed at the boundary between the small and large intestines, with Neocallimastigomycota and Basidiomycota as the dominant phyla. There were significant differences in the abundance of Neocallimastigomycota and Basidiomycota in the foregut and hindgut (*P* < 0.01). The relative abundance of Ascomycota significantly differed in the foregut and hindgut (*P* < 0.05) **(**Fig. 2A; Additional File 2). The relative abundance of microbial species in different gastrointestinal segments differed at the phylum level. Neocallimastigomycota was more abundant in dorsal colon than in the stomach, jejunum, ileum, and caecum (*P* < 0.01, < 0.01, < 0.01, < 0.05, respectively). Basidiomycota was more abundant in the stomach than in the caecum, ventral colon, and dorsal colon (*P* = 0.01, < 0.01 and < 0.01, respectively). Ascomycota was more abundant in the ileum than in the stomach, ventral colon, and dorsal colon (*P* = 0.01, < 0.01, < 0.01, respectively). Chytridiomycota was more abundant in the jejunum than in other parts (all *P* < 0.01) **(**Fig. 2A; Additional File 3).

At the family level, the relative abundance of *Neocallimastigaceae* in the hindgut was significantly higher than that in the foregut (*P* < 0.01) **(**Fig. 2B**)**. At the genus level, the fungal composition of luminal contents changed significantly during the transition from the small intestine to the large intestine. Although the abundance of some fungal genera was high in the foregut, no single genus exceeded 15% in any foregut samples **(**Fig. 2C; Additional File 5). The genera with the most abundant genera detected in the fungal community composition in the hindgut (relative abundance > 1%) included *Cryptococcus* (2.34%), *Cladosporium* (2.36%), and *Alternaria* (1.39%). The large proportion of “other genera” in the hindgut indicated that many genera had not been identified (Additional File 5). Analysis of significant differences among different groups showed that *Cladosporium* was more abundant in the ileum (I) than in the stomach, caecum, ventral colon, and dorsal colon (*P* < 0.01 or *P* < 0.05) **(**Fig. 2C, Additional File 6).

**Fig. 3:**
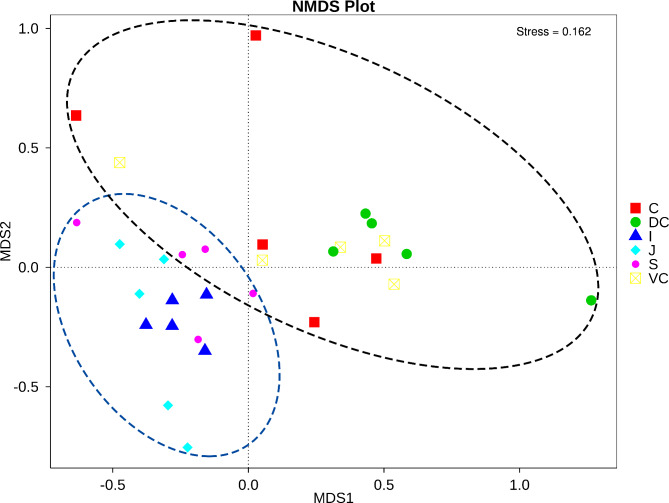
Nonmetric multidimensional scaling (NMDS) with clustering represents the fungal structure dissimilarity found among samples from Mongolian horse GIT compartments. In each figure, S represents the stomach segment, J represents the jejunum segment, I represents the ileum segment, C represents the caecum segment, VC represents the ventral colon segment, and DC represents the dorsal colon segment

To better assess the structural differences between samples, all OTUs were subjected to NMDS analysis (Fig. 3). The samples were formed into two distinct clusters, UG and LG. The LG samples were more dispersed, indicating a greater difference in the fungal community across the segments. In contrast, the UG samples were relatively clustered, indicating higher compositional similarity. LDA and LEfSe were used to further search for biomarkers with statisticant differences between groups [38]. Twenty funfal genera had significant biological differences between different parts of the intestine, and 36 taxa at different taxonomic levels had LDA values greater than 4 (Additional File 7). At the phylum level, the abundance of Basidiomycota was the highest in the stomach, that of Ascomycota was highest in the ileum, and that of Neocallimastigomycota was highest in the dorsal colon compared with those of different parts of the GIT, and the ventral colon did not have a taxon with LDA > 4.

### Functional prediction of the FunGuild gene

**Fig. 4:**
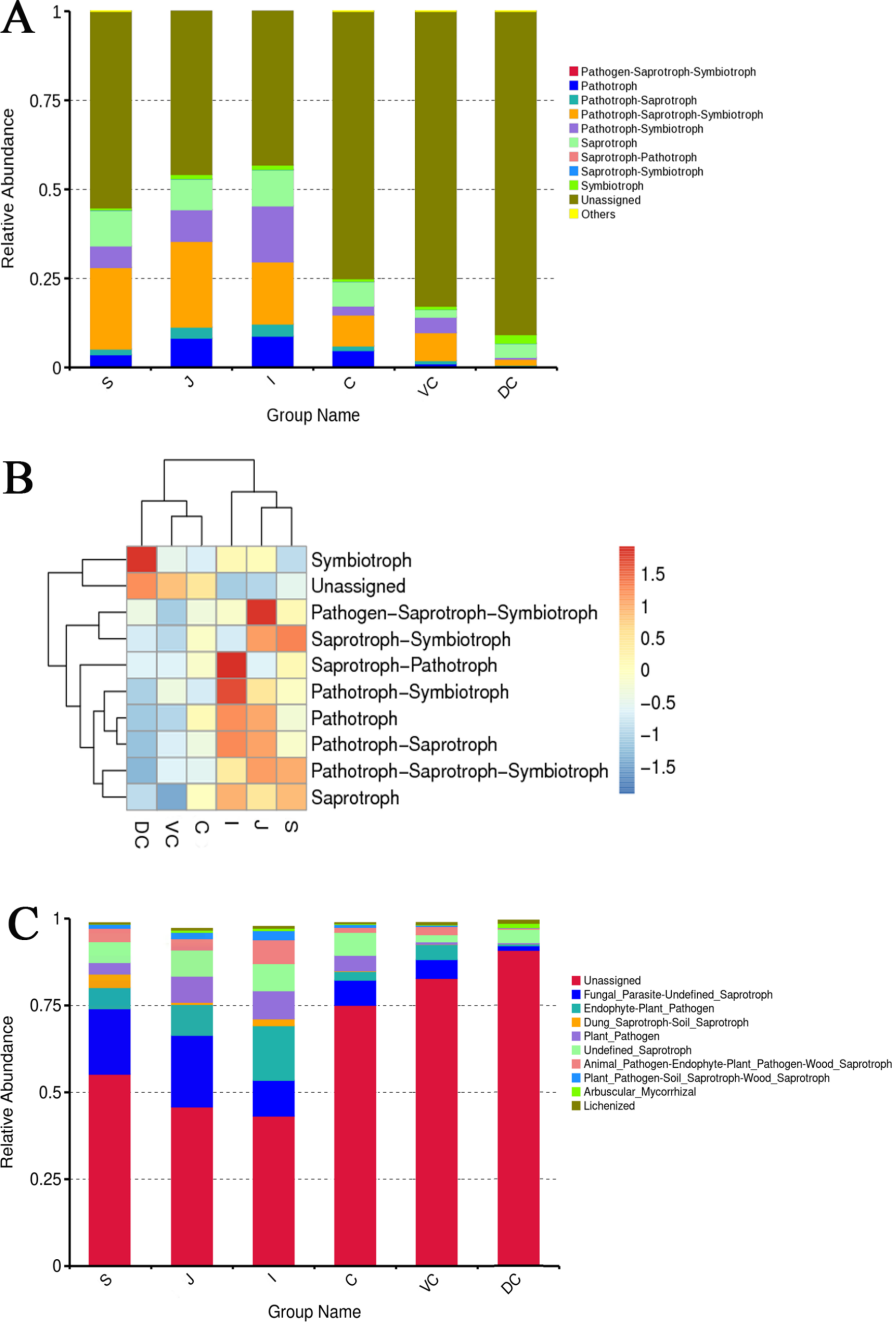
Functional analysis of gastrointestinal fungi in Mongolian equine. **A**: Trophic mode histogram of relative abundance; **B**: Abundance clustering heatmap; **C**: Histogram of relative abundance based on the guilds. In each figure, S represents the stomach segment, J represents the jejunum segment, I represents the ileum segment, C represents the caecum segment, VC represents the ventral colon segment, and DC represents the dorsal colon segment

FUNGuild was used to predict the function of fungal communities in different parts of the equine GIT [39], and based on the trophic mode, fungi were divided into symbiotrophs, saprotrophs, and pathotrophs. A total of 1,587 OTUs in this study were divided into nine nutrient types, and 43 functional groups were detected. The nine main functional groups with high abundance were analysed **(**Fig. 4A**)**. The saprophyte-symbiotic trophic type in the ileum (I) group, pathological saprophyte-symbiotic trophic type in the jejunum (J) and symbiotic trophic type in the dorsal colon (DC) group were significantly higher than in other segments **(**Fig. 4B**)**. The main functional groups of pathotrophic fungi in the GIT of Mongolian horses were animal and plant pathogens. The relative abundance of animal and plant pathogens in the foregut was significantly higher than that in the hindgut (*P* < 0.01), and the highest abundance was observed in the ileum (I) group **(**Fig. 4C**)**.

## Discussion

Compared with traditional culture methods, high-throughput sequencing technology is an efficient, rapid, and accurate approach to analysing microbial community composition, especially for studying microbial species with harsh environmental requirements [40]. At present, studies on equine intestinal microbiota have mostly focused on the bacterial diversity of stool samples [4, 7, 12, 41, 42], but these studies have limitations. Indeed, it should be borne in mind that stool samples can only represent the microbial structure of the distal part of the hindgut (colon and rectum) and cannot truly reflect the composition of the entire gastrointestinal microbial community. In addition, significant emphasis has been placed on the role of fungal diversity in regulating gut microbiota function.

Fungi are an important component of the intestinal flora and affect the balance of intestinal microecology. Simultaneously, they also rely on and harmoniously coexist with gut bacteria to maintain normal intestinal function. In this study, we compared the structure and composition of the fungal community in different parts of the gastrointestinal tract of Mongolian horses by sequencing an internal transcribed spacer region. Currently, studies on the structure and composition of fungi in different parts of the Mongolian horse gut are limited to the analysis of clone libraries [24]. Studies using high-throughput sequencing technology to analyse the whole-gut fungal community and functional prediction in horses have been reported [14], but no systematic studies have been conducted on the whole-gut fungal community diversity in Mongolian horses.

### Fungal community composition and hindgut fermentation characteristics in the gastrointestinal tract of Mongolian horses

The results of the present study showed that the dominant fungal phyla in the GIT of Mongolian horses were Basidiomycota, Ascomycota, and Neocallimastigomycota, which accounted for more than 97% of the total intestinal fungi, consistent with the findings of past literature on the microbial life of Mongolian horse feeces [43]. Basidiomycota and Ascomycota are non-strictly anaerobic fungi. Most fungi belonging to the Ascomycota are saprophytes, which decompose plant residues and degrade soil organic matter [44]. However, most fungi belonging to Basidiomycota have a strong ability to decompose lignocellulose [45]. Neocallimastigomycota plays an important role in the degradation of structural carbohydrates. These three fungal groups are abundant in the GIT of Mongolian horses, which is consistent with the results of a previously conducted study on equine faecal fungal groups [14]. This indicates that Mongolian horses are more adapted to consuming plant food resources, and the metabolic effect accounts for the largest proportion during the prediction of microbial function.

The results of this study revealed that Ascomycota is the dominant phylum in the GIT of Mongolian horses, with a relative abundance of 28.33% (Additional File 8). Saccharomycetes is an important member of Ascomycetes. Reportedly, feeding live yeast to ruminants can significantly increase the abundance of rumen microorganisms and improve production performance [46]. Interestingly, supplementing live yeast to horse diets can reportedly significantly improve feed digestibility and performance, increase feed intake, reduce the number of pathogenic intestinal bacteria, and reduce the negative impact of horse production on the environment [47]. In our study, we found that Neocallimastigomycota represented the dominant group of anaerobic fungi in the hindgut, especially in the dorsal colon, and its relative abundance was significantly higher than that in the foregut (*P* < 0.01). Current evidence suggests that Neocallimastigomycota plays an important role in host cellulose digestion, suggesting that horses are hindgut fermenters. Neocallimastigomycota is mainly found in the digestive tract of animals fed high-fibre vegetative diets. It destroys fibrous diets through highly active cellulolytic enzymes at the tip of the hyphae and mechanical penetration during growth. In turn, it increases the access and possibility of other types of cellulose-degrading microorganisms to obtain carbohydrates. A rich literature substantiates that, although the proportion of anaerobic fungi in intestinal microorganisms is not high, they can release more than 50% of fermentable polysaccharides from plant feed ingested by moving objects [46, 48]. Moreover, in vitro studies have shown that anaerobic fungi can release up to 95% fermentable polysaccharides from plant leaves during the culture period [49]. This study substantiated comparable fungal diversity in different parts of the GIT. The richness index of the hindgut fungal community was significantly higher than that of the foregut. Futhermore, fungi associated with higher abundance were found in the hindgut, which played an important role in cellulose degradation.

At the family level, the relative abundances of *Neocallimastigaceae* and *Tuberaceae* in the hindgut were significantly higher than those in the foregut(*P* < 0.01). *Neocallimastigaceae* is the only obligate anaerobic group of fungi and the only group currently known to contain six genera. At the genus level, the relative abundance of the six known genera of *Neocallimastigaceae* in the hindgut is extremely low, indicating that many genera of *Neocallimastigaceae* are found in the Mongolian horse hindgut, and the role of these genera in the gut is currently unclear, warranting further research. These fungi are not necessarily active in the GIT tract. Like humans, these may be present in their food [50].

### Diversity and disease resistance of fungal communities in the gastrointestinal tract

The proportions of Basidiomycota and Ascomycota were the highest in the foregut contents of Mongolian horses. In humans, the relative abundance of Basidiomycota and Ascomycota is positively associated with the occurrence of inflammatory bowel disease (IBD) and colorectal cancer (CRC) [51, 52]. Therefore, the stability and balance of the fungal community structure in the foregut (especially the stomach and ileum) is an important index directly related to the intestinal health of horses.

At the family level, the relative abundances of Tremellales incertae sedis, *Davidiellaceae*, and *Pleosporaceae* in the foregut were significantly higher than those in the hindgut (*P* < 0.01). However, little is known about the specific role of these family-level fungi in equine physiology and metabolism. At the genus level, the relative abundances of *Cryptococcus*, *Cladosporium*, *Alternaria*, and *Sarocladium* in the foregut were significantly higher than those in the hindgut (*P* < 0.01 or *P* < 0.05). These fungi are opportunistic pathogens in the human body. For example, *Cladosporium* is not only significantly enriched in the gut of subjects with nonalcoholic fatty liver disease (NAFLD) but also correlated with the severity of the disease and can be used as a disease predictor of nonalcoholic steatohepatitis and significant fibrosis. *Cladosporium* is one of the most common fungal genera in the GIT [53] and has been reported to be a human pathogen in recent years [54, 55]. Meanwhile, studies have found that the abundance of *Cladosporium* in the intestinal tract is significantly increased in overweight and obese people. Moreover, it can directly or indirectly participate in the occurrence and development of inflammatory bowel disease and HIV infection through interaction with bacteria [56, 57]. Fusarium is also an opportunistic pathogen whose abundance is significantly increased in alcoholic liver disease and has been implicated in the progression of inflammatory bowel disease (IBD) and acquired immunodeficiency syndrome (AIDS) [58]. In short, Mongolian horses can still maintain good health and survive in harsh environments all year long despite the abundance of saprophytic and pathogenic fungi in their foregut, substantiating that Mongolian horses have strong disease resistance.

### Fungal community diversity in different gastrointestinal tract segments

We found that Neocallimastigomycota and Basidiomycota were predominantly found in the contents of the hindgut of Mongolian horses, which is inconsistent with the results of studies on intestinal microbial communities of horses in faecal samples [43]. This finding suggests that stool may not fully represent the composition of the hindgut microbiome, or it may be related to the sequencing method (DNA extraction technology and sequencing platform), breed, geographical location, or diet. Indeed, further studies are needed to verify the relevance of these factors.

The composition of the fungal community in the contents of different parts of the Mongolian horses’ GIT was significantly different. The ecological environment in the whole intestinal tract was not unchanged but showed significant regional changes. Moreover, the fungal community in the GIT of Mongolian horses could be divided into two distinct regions, the foregut and the hindgut, consistent with our previous study on the bacterial diversity in the GIT of Mongolian horses. While the different parts of the foregut had similar fungal communities, those of the hindgut fungal community were highly variable among the specific parts, as well as in different horses. The NMDS plot shows that individual horses differed most in the caecum fungal community. This also indicates that there may be an interaction between different microbial or fungal groups, which jointly maintain the intestinal microbial balance and body health of Mongolian horses.

### Functional analysis of fungal composition in the gastrointestinal tract

There are significant differences in fungal function between the foregut and hindgut in horses, and they were confirmed by FUNGuild prediction of fungal community function in the GIT of horses. The functional analysis results were consistent with the results of fungal diversity analysis in different parts. We found that fungi in the foregut of Mongolian horses were mainly pathologically nutritive and contained a large number of animal and plant pathogens, especially in the small intestine (jejunum and ileum), indicating that the foregut may be the most important immune site in the digestive system of Mongolian horses, which accounted for the high disease resistance of Mongolian horses. The unassigned functional group in the hindgut was significantly higher than that in the foregut, indicating that the functions of fungal groups in the hindgut have not been fully explored, and further research is warranted. The FUNGuild function prediction and analysis methods still have some limitations. In the future, metagenomic sequencing, related functional gene analysis, metabolome sequencing, and other methods should be combined to more accurately analyse and grasp the function of the Mongolian horse intestinal fungal community. Mongolian horses have strong adaptability and disease resistance, which is related to their excellent breed characteristics and long-term grazing and foraging, which may promote the formation of unique intestinal microbiota.

This study has several limitations, which decrease the robustness of our findings. First, compared with whole-genome shotgun sequencing, ITS amplicon sequencing has limited resolution and can only be annotated to the family or genus level. In addition, given the lack of fungal information in omics databases, less than half of the genes can be annotated after fungal sequencing. Moreover, many bacteria exist in the digestive tract microbiota, which increases the difficulty of obtaining sufficient fungal genomes in the digestive tract microbiota. Finally, the limited sample size in this study may have reduced the efficiency of detecting differences between gastrointestinal regions to some extent. Although this study provides a detailed composition of Mongolian horses’ entire gastrointestinal fungal flora, more studies are needed to determine the influence of other factors, such as age, geographical location, and diet.

## Conclusion

Based on ITS amplicon high-throughput sequencing and FUNGuild function prediction, this study provides novel insights into fungal communities and functions in different parts of the digestive tract in healthy Mongolian horses. The following conclusions were obtained: (1) the Mongolian horse is a hindgut fermentative animal, which is related to its unique fungal community structure; (2) there are a large number of pathogenic fungi in the foregut of Mongolian horses, indicating that Mongolian horses have strong disease resistance; and (3) based on the composition and function of fungal groups, the digestive tract of Mongolian horses can be divided into the foregut and hindgut.

### Electronic supplementary material

Below is the link to the electronic supplementary material.

Supplementary Material 1

## Data Availability

The datasets generated and analyzed during the current study are available in the NCBI Sequence Read Archive at https://www.ncbi.nlm.nih.gov/bioproject/PRJNA898862.

## List of abbreviations

GIT: Gastrointestinal tracts
UG: Upper GIT
LG: Lower GIT
