# Supplementary material for: Diversity and functional prediction of fungal communities in different segments of mongolian horse gastrointestinal tracts
Source: BMC Microbiol. 2023 Sep 9;23:253. doi: 10.1186/s12866-023-03001-w (PMC10492400; doi:10.1186/s12866-023-03001-w)

**[Additional File 1](https://static-content.springer.com/esm/art:10.1186/s42523-022-00192-x/MediaObjects/42523_2022_192_MOESM1_ESM.pdf).** OTUs clustering and annotation statistics.


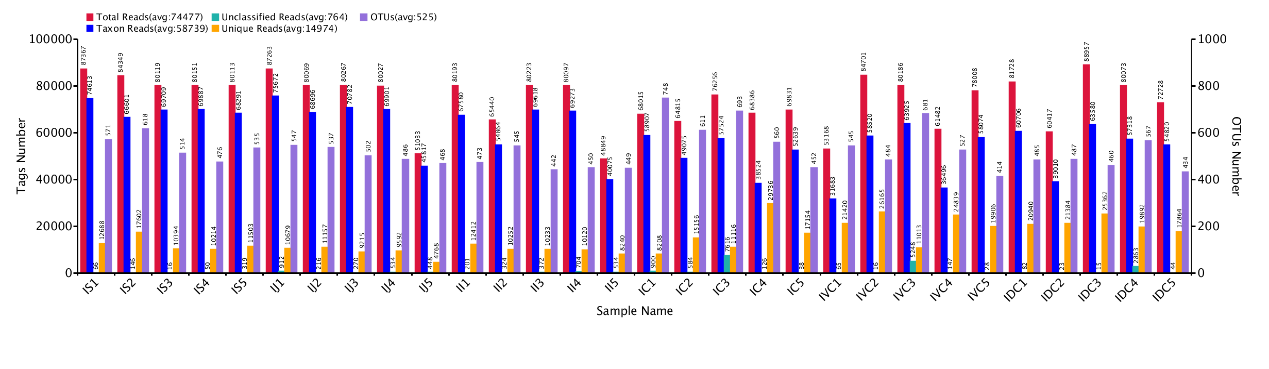


Abscissa: sample name; First ordinate (reads number): reads Number; Total reads (red): the Total number of reads per sample (valid data for subsequent analysis such as OTUs clustering per sample); Taxon reads (blue): Number of reads used to build OTUs and obtain annotated information; Unique reads (orange): refers to the number of reads with frequency 1 that cannot be clustered into OTUs (sequences that cannot be clustered into OTUs will not be used for subsequent analysis). The second ordinate (OTUs Number): refers to the number of OTUs; OTUs (purple): indicate the number of OTUs obtained for each sample; Unclassified reads (tea green): refer to the number of reads without any classified information.

**[Additional File 2](https://static-content.springer.com/esm/art:10.1186/s42523-022-00192-x/MediaObjects/42523_2022_192_MOESM1_ESM.pdf).** Breed comparisons for the relative abundance of LG and UG  fungal composition at the phylum level.

| **Phylum** | **The upper GIT** | **The lower GIT** |
| --- | --- | --- |
| Neocallimastigomycota | 0.1966±0.5488^B^ | 0.5384±0.3786^A^ |
| Basidiomycota | 0.6002±0.1726^A^ | 0.22914±0.2471^B^ |
| Ascomycota | 0.3619±0.1628^a^ | 0.2047±0.1802^b^ |
| Glomeromycota | 0.0047±0.0059 | 0.0054±0.0138 |
| Zygomycota | 0.0051±0.0064 | 0.0028±0.0030 |
| Chytridiomycota | 0.0030±0.0042 | 0.0008±0.0011 |

Note: Different uppercase letters mean the significance at *P* < 0.01, Different lowercase letters mean the significance at *P* < 0.05 and blank indicate no significant difference *P* > 0.05.

**[Additional File 3](https://static-content.springer.com/esm/art:10.1186/s42523-022-00192-x/MediaObjects/42523_2022_192_MOESM1_ESM.pdf).** Comparison of relative abundance of gastrointestinal fungi at the phylum level.

| Phylum | Stomach（S） | Jejunum（J） | Ileum（I） | Cecum（C） | Ventral colon（VC） | Dorsal colon（DC） |
| --- | --- | --- | --- | --- | --- | --- |
| Neocallimastigomycota | 0.0466±0.0956^C^ | 0.0041±0.0013C | 0.0083±0.0058^C^ | 0.2491±0.2817^Bb^ | 0.5731±0.4080^ABa^ | 0.7931±0.2620^A^ |
| Basidiomycota | 0.7140±0.1908^A^ | 0.6140±0.1406^A^ | 0.4727±0.1064^A^ | 0.3757±0.2619^Ba^ | 0.2474±0.2850^Bb^ | 0.0644±0.0.0520^C^ |
| Ascomycota | 0.2347±0.1341^Ab^ | 0.3560±0.1398^Aa^ | 0.4949±0.1137^Aa^ | 0.3376±0.1727^Aa^ | 0.1589±0.1500^B^ | 0.1175±0.1647^B^ |
| Glomeromycota | 0.0007±0.0004 | 0.0064±0.0080 | 0.0070±0.0051 | 0.0024±0.0013 | 0.0015±0.0010 | 0.0124±0.0241 |
| Zygomycota | 0.0019±0.0025 | 0.0057±0.0043 | 0.0077±0.0010 | 0.004±0.0043 | 0.0016±0.0013 | 0.0022±0.0020 |
| Chytridiomycota | 0.0005±0.0007^B^ | 0.0067±0.0056^A^ | 0.0019±0.0013^B^ | 0.0011±0.0084^B^ | 0.0011±0.0017^B^ | 0.0004±0.0006^B^ |

Note: Different uppercase letters mean the significance at *P* < 0.01, Different lowercase letters mean the significance at *P* < 0.05 and blank indicate no significant difference *P* > 0.05.

**[Additional File 4](https://static-content.springer.com/esm/art:10.1186/s42523-022-00192-x/MediaObjects/42523_2022_192_MOESM1_ESM.pdf). OTUs Venn diagram of intestinal flora in Mongolian horses.** In each figure, C represents the cecum segment, VC represents the ventral colon segment, and DC represents the dorsal colon segment.

**
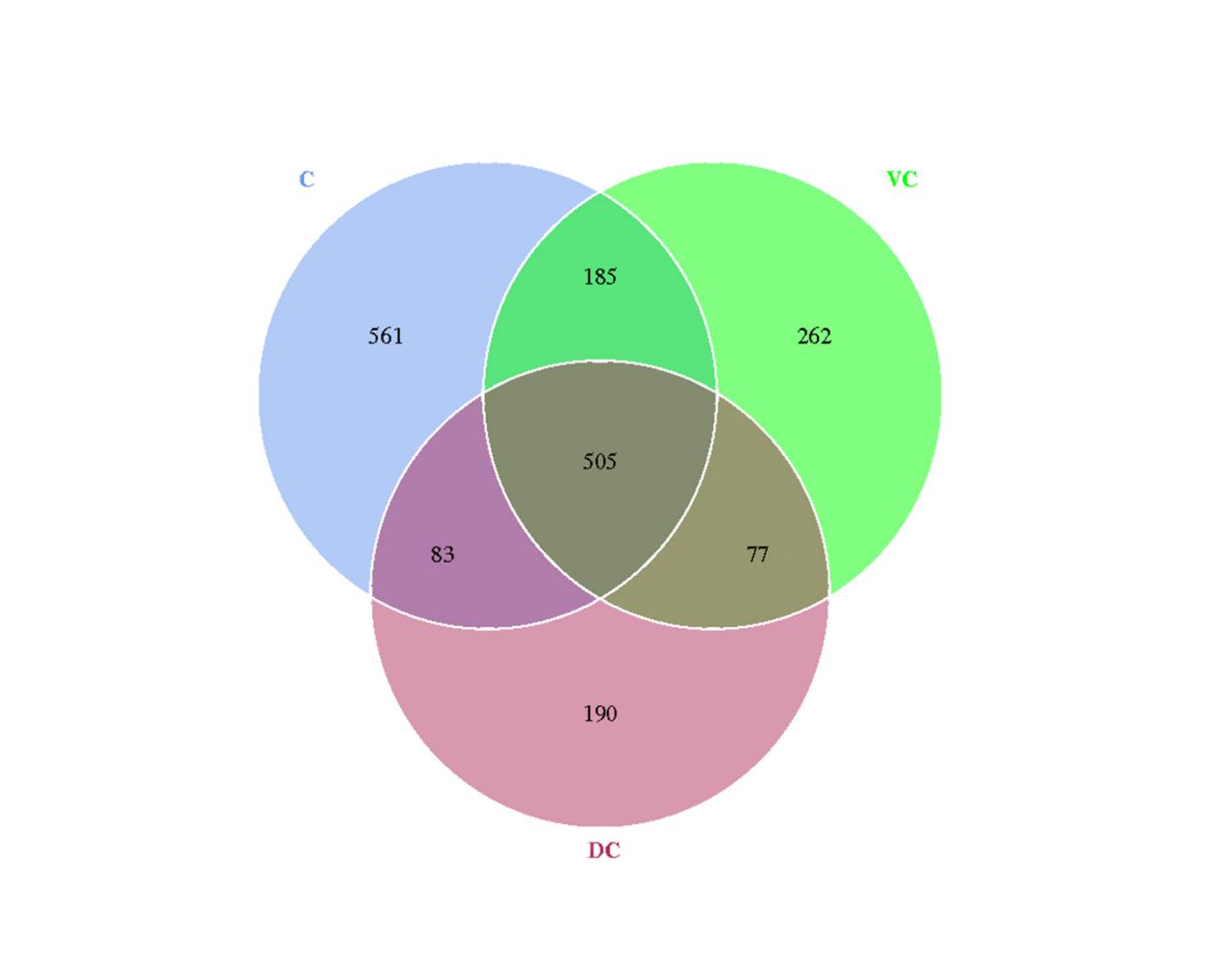
**

**[Additional File 5](https://static-content.springer.com/esm/art:10.1186/s42523-022-00192-x/MediaObjects/42523_2022_192_MOESM1_ESM.pdf).** Comparisons for the relative abundance of UG and LG microbiota at the genus level.

| Genus | The upper GIT | The lower GIT |
| --- | --- | --- |
| *Cryptococcus* | 0.1154±0.1520^a^ | 0.0234±0.0361^b^ |
| *Cladosporium* | 0.0966±0.077^A^ | 0.0236±0.0340^B^ |
| *Lysurus* | 0.0215±0.0483 | 0.0010±0.0014 |
| *Alternaria* | 0.0465±0.0350^A^ | 0.0139±0.0183^B^ |
| *Gibberella* | 0.0184±0.0158 | 0.0095±0.0299 |
| *Bipolaris* | 0.0137±0.0298 | 0.0011±0.0011 |
| *Candida* | 0.0012±0.0017 | 0.0083±0.0223 |
| *Bullera* | 0.0206±0.0255 | 0.0064±0.0085 |
| *Sarocladium* | 0.0212±0.0224^A^ | 0.0030±0.0040^B^ |
| *Hannaella* | 0.0215±0.0212 | 0.0094±0.0114 |
| *others* | 0.5398±0.1770^B^ | 0.8554±0.1372^A^ |

Note: Different uppercase letters mean the significance at *P* < 0.01, Different lowercase letters mean the significance at *P* < 0.05 and blank indicate no significant difference *P* > 0.05.

**[Additional File 6](https://static-content.springer.com/esm/art:10.1186/s42523-022-00192-x/MediaObjects/42523_2022_192_MOESM1_ESM.pdf).** Comparison of relative abundance of GIT fungi at the genus level.

| Genus | Stomach（S） | Jejunum（J） | Ileum（I） | Cecum（C） | Ventral colon（VC） | Dorsal colon（DC） |
| --- | --- | --- | --- | --- | --- | --- |
| *Cryptococcus* | 0.1251±0.1701 | 0.1459±0.1756 | 0.0751±0.1332 | 0.0448±0.0591 | 0.0181±0.0114 | 0.0072±0.0024 |
| *Cladosporium* | 0.0575±0.0293^Ab^ | 0.0810±0.0526^Aa^ | 0.1511±0.1045^Aa^ | 0.0242±0.0191^B^ | 0.0419±0.0530^B^ | 0.0047±0.0023^B^ |
| *Lysurus* | 0.0389±0.0843 | 0.0061±0.0056 | 0.0196±0.01833 | 0.0018±0.0023 | 0.0008±0.0003 | 0.0005±0.0001 |
| *Alternaria* | 0.0385±0.0158^Aa^ | 0.0324±0.0205^Aa^ | 0.0686±0.5180^Aa^ | 0.0143±0.0091^B^ | 0.0236±0.0290^Ab^ | 0.0037±0.0012^B^ |
| *Gibberella* | 0.0115±0.0123 | 0.0194±0.0147 | 0.0244±0.0201 | 0.0262±0.0511 | 0.0018±0.0018 | 0.0006±0.0003 |
| *Bipolaris* | 0.0012±0.0010 | 0.0153±0.0297 | 0.0247±0.0433 | 0.0014±0.0010 | 0.0014±0.0015 | 0.0004±0.0001 |
| *Candida* | 0.0005±0.0003 | 0.0020±0.0028 | 0.0012±0.0007 | 0.0045±0.0055 | 0.0016±0.0015 | 0.0188±0.0387 |
| *Bullera* | 0.0224±0.0339 | 0.0245±0.0286 | 0.0148±0.0157 | 0.0080±0.0069 | 0.0100±0.0124 | 0.0011±0.0004 |
| *Sarocladium* | 0.0154±0.0140^a^ | 0.0230±0.0309^a^ | 0.0251±0.0226^a^ | 0.0055±0.0051^a^ | 0.0029±0.0039^b^ | 0.0008±0.0006^b^ |
| *Hannaella* | 0.0324±0.0293^Aa^ | 0.0224±0.0167^Ab^ | 0.0097±0.0106^Aa^ | 0.0120±0.0076^Aa^ | 0.0149±0.0166^Aa^ | 0.0015±0.0008^B^ |
| *others* | 0.5858±0.1894^Bb^ | 0.5335±0.2331^B^ | 0.5004±0.1218^B^ | 0.7859±0.1520^Aa^ | 0.8504±0.1473^A^ | 0.9298±0.0897^A^ |

Note: Different uppercase letters mean the significance at *P* < 0.01, Different lowercase letters mean the significance at *P* < 0.05 and blank indicate no significant difference *P* > 0.05.

**[Additional File 7](https://static-content.springer.com/esm/art:10.1186/s42523-022-00192-x/MediaObjects/42523_2022_192_MOESM1_ESM.pdf).** LDA value distribution histogram (only the genera LDA scores above 4 are shown).


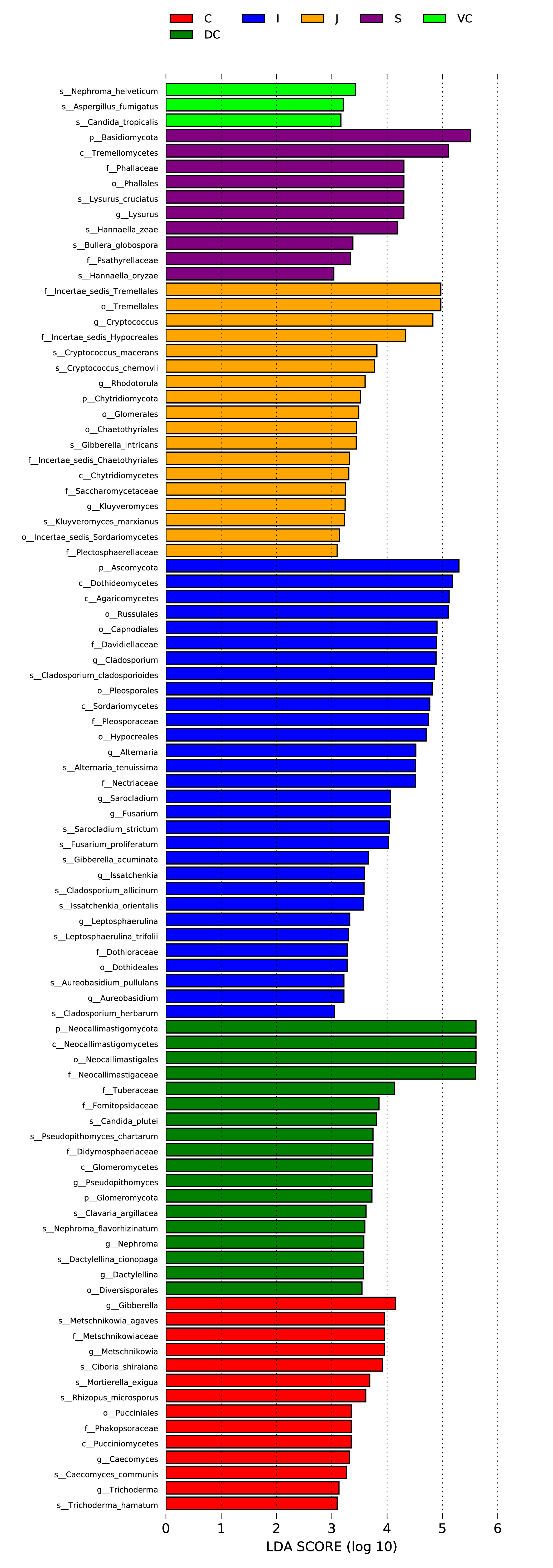


**[Additional File 8](https://static-content.springer.com/esm/art:10.1186/s42523-022-00192-x/MediaObjects/42523_2022_192_MOESM1_ESM.pdf).** Distribution of the Mongolian horses GIT fungal community composition.


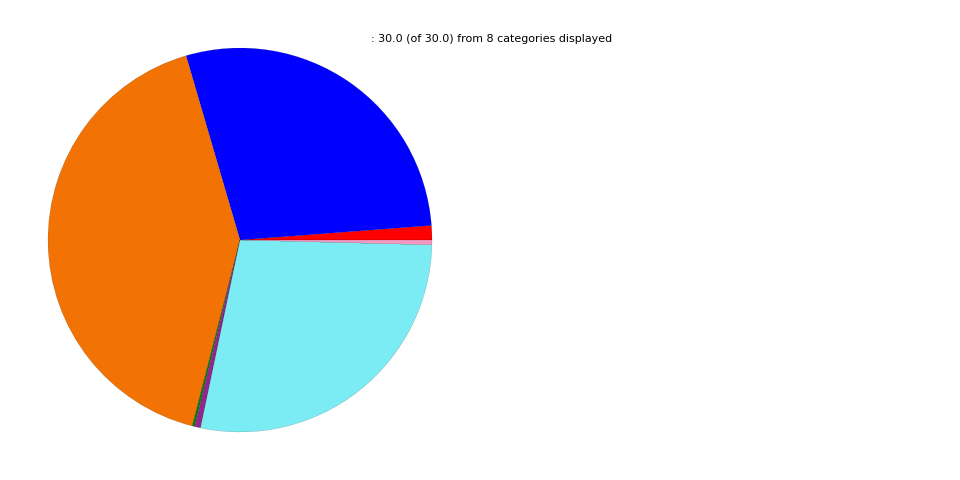

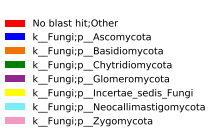

Supplement: Supplementary file 1 — Supplementary Material 1 [file 12866_2023_3001_MOESM1_ESM.docx]
